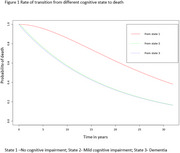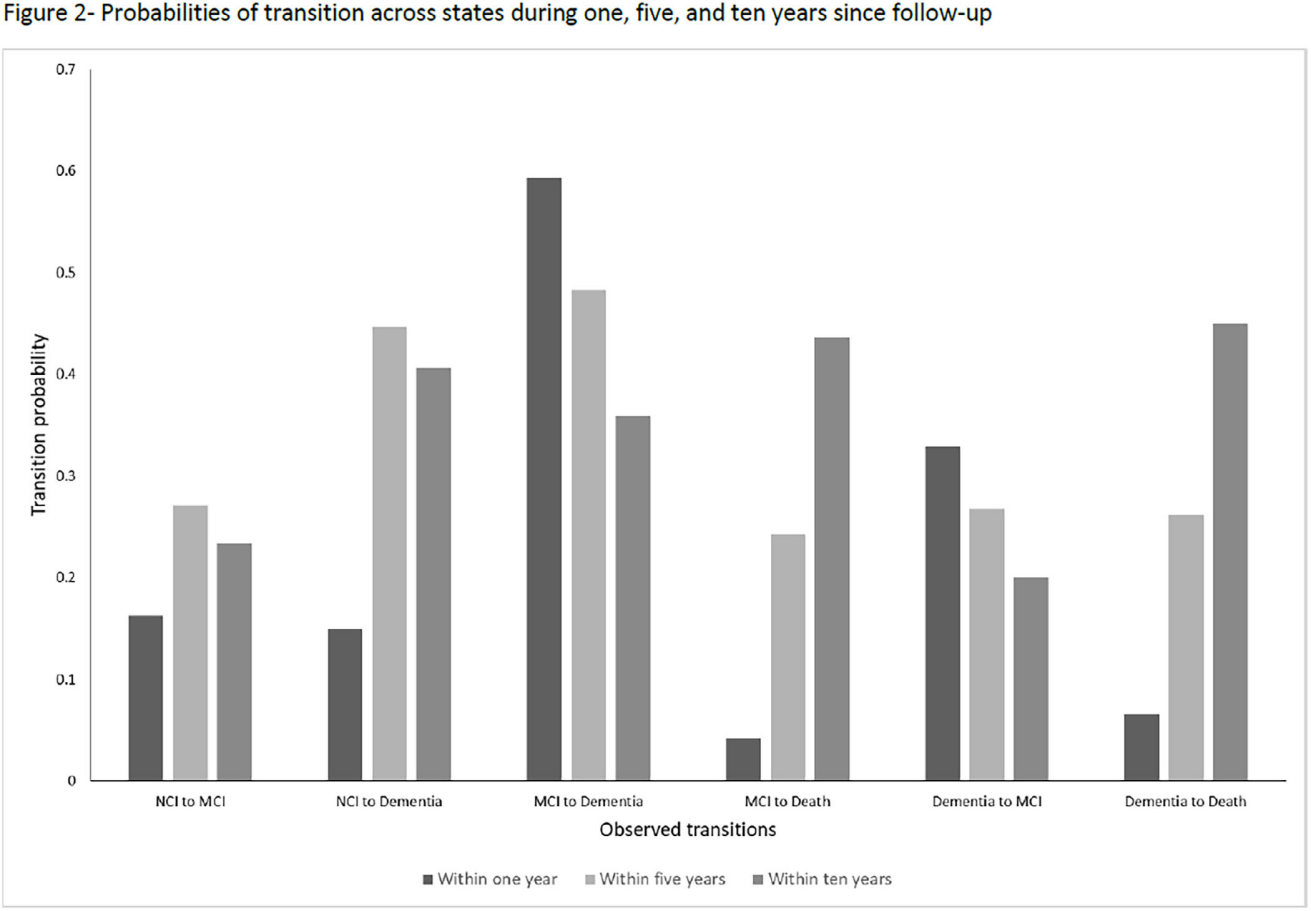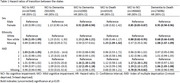# Transitions through different cognitive states and death in dementia in the UK population

**DOI:** 10.1002/alz70860_105836

**Published:** 2025-12-23

**Authors:** Subhashisa Swain, Clare Bankhead, Rafael Perera, Klaus P Ebmeier

**Affiliations:** ^1^ University of Oxford, Oxford, Oxford, United Kingdom; ^2^ Keele University, Keele, Stoke on Trent, United Kingdom; ^3^ Wellcome Centre for Integrative Neuroimaging, University of Oxford, Oxford, United Kingdom

## Abstract

**Background:**

Nearly 900,000 people in the UK live with dementia (1), yet transitions between cognitive states and death remain poorly understood. This study explores the influence of sex, ethnicity, and socioeconomic status on transitions across no cognitive impairment (NCI), mild cognitive impairment (MCI), dementia, and death.

**Method:**

We analysed electronic health records from the UK Clinical Practice Research Datalink (2000–2022), including 668,554 adults diagnosed with dementia or MCI. Participants were followed from the 10 years before their first recorded dementia or MCI diagnosis, tracking transitions to MCI until death or moved out of the records. Data on sex, ethnicity, and socioeconomic status (indexed by multiple deprivation) were collected. Transition hazard ratios (HRs) and time probabilities in both directions were estimated using a multistate Markov model in R.

**Result:**

Among 668,554 individuals, 423,715 (63.4%) had MCI, 633,505 (94.2%) had dementia, and 317,415 (47.4%) died. Nearly 62% were women, and the average age at dementia diagnosis was 81 years (standard deviation 9 years). The average duration a person stayed in NCI, MCI, and dementia state were 2.7 years, 5 months, and 8 months, respectively. Death rates were lower from NCI than from MCI or dementia. Women showed reduced risks of transitioning from MCI to dementia (HR 0.91, 95% CI 0.89–0.94), MCI to NCI (HR 0.85, 95% CI 0.83–0.87), and dementia to death (HR 0.95, 95% CI 0.94–0.96) compared to men. Non‐white populations had higher risks of transitioning from NCI to MCI (HR 1.06, 95% CI 1.03–1.09) and MCI to dementia (HR 1.13, 95% CI 1.06–1.21) but lower risks for dementia to death and MCI to NCI (reserve transition) compared to white populations. Higher socioeconomic status correlated with increased transitions from MCI to dementia and MCI to NCI. Transition probabilities from MCI to dementia or dementia to death rose over time while reverse transitions declined. The likelihood of moving from NCI to MCI or MCI to dementia peaked at 5 years.

**Conclusion:**

Sex, ethnicity, and socioeconomic status influence cognitive trajectories in the UK. The substantial probabilities of reverse transitions, such as dementia to MCI and MCI to NCI, highlight areas for further exploration.